# Supplementary material for: Transcriptomic Analysis of Neocaridina denticulata sinensis Gills Following FPPS Knockdown Reveals Its Regulatory Role in Immune Response
Source: Int J Mol Sci. 2024 Dec 25;26(1):65. doi: 10.3390/ijms26010065 (PMC11720513; doi:10.3390/ijms26010065)
Supplement: Supplementary file 1 [file ijms-26-00065-s001.zip › ijms-3328490-supplementary.pdf]

Transcriptomic Analysis of *Neocaridina denticulata sinensis* Gills Following FPPS Knockdown Reveals Its Regulatory Role in Immune Response

Hongrui Li [1,2,†], Dandan Feng [1,†], Chunyu Zhang [1,2], Mengfei Liu [1,2], Zixuan Wu [1,2], Yuke Bu [1], Jiquan Zhang [1,*] and Yuying Sun [1,2,*]

[1] School of Life Sciences, Hebei Basic Science Center for Biotic Interaction, Hebei University, Baoding 071002, China; hongruili2024@163.com (H.L.); feng_18245151652@163.com (D.F.); chunyuzhang0124@163.com (C.Z.); 13230612530@163.com (M.L.); zxwu0527@163.com (Z.W.); b18453701822@163.com (Y.B.)

[2] Institute of Life Science and Green Development, Hebei University, Baoding 071002, China

* Correspondence: zhangjiquan@hbu.edu.cn (J.Z.); sunyuying125@hbu.edu.cn (Y.S.)

† These authors contributed equally to this work.

Supplementary Materials Table S1. Gene sequences mentioned in this paper.

Gene names	Species	Gene ID
PchFPPS	*Penaeus chinensis*	LOC125037521
PmFPPS	*Penaeus monodon*	LOC119586416
PjFPPS	*Penaeus japonicus*	LOC122246647
PvFPPS	*Penaeus vannamei*	LOC113823975
PclFPPS	*Procambarus clarkii*	LOC123767848
EsFPPS-1	*Eriocheir sinensis*	LOC127002236
EsFPPS-2	*Eriocheir sinensis*	LOC127002260
PtFPPS	*Portunus trituberculatus*	LOC123515367
HaFPPS	*Homarus americanus*	LOC121872984
MrFPPS	*Macrobrachium rosenbergii*	LOC136851923
SpFPPS	*Scylla paramamosain*	LOC135109140
CqFPPS	*Cherax quadricarinatus*	LOC128690491
CeFPPS	*Caenorhabditis elegans*	173075
DrFPPS	*Danio rerio*	552997
BmFPPS-1	*Bombyx mori*	100101207
BmFPPS-2	*Bombyx mori*	100101208
DmFPPS	*Drosophila melanogaster*	36209

Note: The *FPPS* genes of different species include *P. chinensis* (*PchFPPS*), *P. monodon* (*PmFPPS*), *P. japonicus* (*PjFPPS*), *P. vannamei* (*PvFPPS*), *P. clarkii* (*PclFPPS*), *E. sinensis* (*EsFPPS-1, EsFPPS-2*), *P. trituberculatus* (*PtFPPS*), *H. americanus* (*HaFPPS*), *M. rosenbergii* (*MrFPPS*), *S. paramamosain* (*SpFPPS*), *C. quadricarinatus* (*CqFPPS*), *C. elegans* (*CeFPPS*), *D. rerio* (*DrFPPS*), *B.mori* (*BmFPPS-1*, *BmFPPS-2*), *D. melanogaster* (*DmFPPS*).

Supplementary Materials Table S2. Species genome data mentioned in this paper.

Species	BioProject
P. chinensis	PRJNA691453
P. monodon	PRJNA611030
P. japonicus	PRJDB11151
P. vannamei	PRJNA438564
P.s clarkii	PRJNA727411
E. sinensis	PRJNA737102
P. trituberculatus	PRJNA555262
H. americanus	PRJNA655509
M. rosenbergii	PRJNA1126456
C. quadricarinatus	PRJNA905544
S. paramamosain	PRJNA1059155

Supplementary Materials Table S3. Summary of data statistics by Illumina sequencing.

Sample	Raw Reads	Clean Reads	Raw Bases (bp)	Clean Bases (bp)	Q20 (%)	Q30 (%)
C12S1	52,221,608	52,207,766	7,833,241,200	7,768,347,570	97.65	93.33
C12S2	44,615,126	44,603,710	6,692,268,900	6,645,628,504	97.69	93.28
C12S3	46,880,400	46,859,650	7,032,060,000	6,963,612,898	97.90	93.89
E12S1	47,258,528	47,033,274	7,088,779,200	6,975,956,769	97.68	93.24
E12S2	44,516,068	44,321,696	6,677,410,200	6,571,515,416	97.69	93.25
E12S3	44,216,278	44,321,696	6,632,441,700	6,571,515,416	97.69	93.25

Supplementary Materials Table S4. FastQC summary statistics for sequencing data.

Sample	Total Sequences	Average Sequence Length	Median Sequence Length	Percentage of Duplicates	Percentage of GC Content
C12S1_1	26,110,804	150	150	67.79%	40%
C12S1_2	26,110,804	150	150	67.60%	40%
C12S2_1	22,307,563	150	150	67.08%	39%
C12S2_2	22,307,563	150	150	66.68%	39%
C12S3_1	23,440,200	150	150	60.60%	40%
C12S3_2	23,440,200	150	150	60.93%	40%
E12S1_1	23,629,264	150	150	81.99%	37%
E12S1_2	23,629,264	150	150	81.25%	37%
E12S2_1	22,258,034	150	150	77.43%	37%
E12S2_2	22,258,034	150	150	77.35%	37%
E12S3_1	21,280,948	150	150	73.50%	39%
E12S3_2	21,280,948	150	150	72.87%	39%

Supplementary Materials Table S5. Primers mentioned in the paper.

Primers	Sequences (5′ - 3′)	Application
dsNdFPPS-F	TAATACGACTCACTATAGGGCGTCTCCTGATCTCCTTA	dsRNA synthesis
dsNdFPPS-R	TAATACGACTCACTATAGGGCTGTATTTCTTATCCCCG	dsRNA synthesis
dsEGFP-F	TAATACGACTCACTATAGGGCAGTGCTTCAGCCGCTACCC	dsRNA synthesis
dsEGFP-R	TAATACGACTCACTATAGGGAGTTCACCTTGATGCCGTTCTT	dsRNA synthesis
RAS P21 Protein Activator 1-F	TCCTTAATCCTAGACAGTTCAACT	qRT-PCR
RAS P21 Protein Activator 1-R	TAAGGCTCCTTGCCACCAAACTCA	qRT-PCR
Coagulation Factor XIII A Chain-F	GTCCTCAGTGACTCGGGACTTATC	qRT-PCR
Coagulation Factor XIII A Chain-R	GTGACATCCTACAGACTGTATTGA	qRT-PCR
Heat Shock Protein 90 Alpha Family Class B Member 1-F	ATCTGGTAAAGAAATCTCTAGAAC	qRT-PCR
Heat Shock Protein 90 Alpha Family Class B Member 1-R	TTCTTGCGATTGGTGGAGTCTTCA	qRT-PCR
Cytochrome P450 Family 307 Subfamily A Member 1-F	AAGTTCGACTTCATCTTCGAAGAC	qRT-PCR
Cytochrome P450 Family 307 Subfamily A Member 1-R	TCCAAGATGAATTTTCTGATGTCA	qRT-PCR
Nicotinamide Phosphoribosyltransferase-F	CAGTAGCATCAAACTCCAGAATAC	qRT-PCR
Nicotinamide Phosphoribosyltransferase-R	TTGCTGCACTCTCAACTGAAGATA	qRT-PCR
Protein Arginine Methyltransferase 8-F	GGAACTTGCAGGTTCTGTCAATAG	qRT-PCR
Protein Arginine Methyltransferase 8-R	CTTAAGCTTCTGAGATTTCTGACA	qRT-PCR
Solute Carrier Family 2 Member 8-F	TATTCCACTGGACAACAGTAGTTC	qRT-PCR
Solute Carrier Family 2 Member 8-R	CCTCGATTTTCCCATGTTGCAGTA	qRT-PCR
18S rRNA-F	GGGGAGGTAGTGACGAAAAAT	qRT/Semi-quantitative PCR
18S rRNA-R	TATATGCTATTGGAGCTGGAA	qRT/Semi-quantitative PCR
Semi-NdFPPS-F	CTGGTATCACCGACAGAGAACTTC	Semi-quantitative PCR
Semi-NdFPPS-R	TCTGTGCCAACCTTACCAGTAACA	Semi-quantitative PCR

Note: T7 promoter sequences are underlined.
